# Infant fungal communities: current knowledge and research opportunities

**DOI:** 10.1186/s12916-017-0802-z

**Published:** 2017-02-13

**Authors:** Tonya L. Ward, Dan Knights, Cheryl A. Gale

**Affiliations:** 1 Biotechnology Institute, University of Minnesota, Saint Paul, MN USA; 2 Department of Computer Science and Engineering, University of Minnesota, Minneapolis, MN USA; 3 Department of Pediatrics, University of Minnesota, 2450 Riverside Ave, Minneapolis, MN 55454 USA

**Keywords:** Mycobiome, Fungi, Microbiome, Bacteria, Infant, Microbiota, Mycobiota, Development

## Abstract

The microbes colonizing the infant gastrointestinal tract have been implicated in later-life disease states such as allergies and obesity. Recently, the medical research community has begun to realize that very early colonization events may be most impactful on future health, with the presence of key taxa required for proper immune and metabolic development. However, most studies to date have focused on bacterial colonization events and have left out fungi, a clinically important sub-population of the microbiota. A number of recent findings indicate the importance of host-associated fungi (the mycobiota) in adult and infant disease states, including acute infections, allergies, and metabolism, making characterization of early human mycobiota an important frontier of medical research. This review summarizes the current state of knowledge with a focus on factors influencing infant mycobiota development and associations between early fungal exposures and health outcomes. We also propose next steps for infant fungal mycobiome research, including longitudinal studies of mother–infant pairs while monitoring long-term health outcomes, further exploration of bacterium–fungus interactions, and improved methods and databases for mycobiome quantitation.

## Background

The beneficial role of microbial colonization to human health is becoming increasingly clear. Recent efforts to define a healthy microbiota show that the microbial communities inhabiting our bodies are diverse and complex, and that colonization dynamics during early life may have lasting impacts on adult health [1]. The term “microbiome” describes the community of microbes living on and within an organism using genetic analysis, usually within a particular niche or body site. Most literature discussing the microbiome, however, pertains only to the bacterial microbiota. Although bacteria constitute the majority of the non-host biomass of humans, they are not the only microorganisms contributing to the microbial ecosystem of the host. For example, human-associated fungi have been largely overlooked. On a cellular basis, approximately 0.1% of the microbes in the adult intestine are fungi, and these fungi are estimated to represent approximately 60 unique species [2, 3]. Although fungi can be human pathogens, especially in association with underlying immunodeficiencies, many fungi are benign commensal inhabitants of human body niches and some have been shown to confer health benefits. For example, previous culture-based and targeted PCR approaches for characterizing the mycobiota have shown humans to be colonized with commensal fungi across multiple body sites [4, 5]. Some species, such as *Saccharomyces boulardii*, have been shown to be effective at preventing and treating human gastrointestinal (GI) diseases (e.g., diarrhea, inflammatory bowel disease, irritable bowel syndrome) [6, 7]. Importantly, overgrowth of fungi leading to infections is more common in infants than adults and can result in significant morbidity and mortality in at-risk infants such as those born prematurely [8–10]. Thus, knowing what a healthy fungal microbiota (mycobiota) is composed of and what factors affect its establishment and maturation during infancy is important if we are to learn how early-life microbial communities affect pediatric and adult health.

With the advent of next-generation sequencing and introduction of low-cost bacterial community profiling approaches, such as 16S rDNA amplicon sequencing, bacterial microbiomes can now be more easily and quickly characterized than ever before. In contrast, development of robust methods to characterize human-associated mycobiota has lagged behind that for bacterial community characterization due to difficulties in sequencing low human-associated fungal biomass and fungal cultivation issues preventing reference sequence generation [11, 12]. Until recently, the majority of published fungal microbiota analyses have relied upon culturing fungi, a method that is less sensitive than sequencing-based approaches [11, 12]. Because fungi are much less abundant than bacteria in most, if not all, human niches, a shotgun metagenomic sequencing approach for the characterization of fungal communities has not been productive. As such, PCR amplicon-based sequencing approaches have been developed and continue to be refined for mycobiome analyses. Genomic targets that have been utilized include the 18S and 28S rDNA sequences, and the internal transcribed spacer regions (ITS1 and ITS2) of the rDNA locus [12]. The ITS regions of the fungal genome are highly variable and capable of providing identification at the species level, but taxonomic characterization of fungi remains challenging as the reference databases available for fungi are far from comprehensive, with up to 20% of sequences annotated incorrectly [13]. Thus, mycobiome characterization may be biased by the marker gene region sequenced and the reference database used. Until improved reference databases are available and new marker gene sequences that more universally distinguish fungi are identified, methods combining broad DNA amplicon surveys with a targeted approach, such as quantitative PCR and/or culturing, will likely be needed to gain robust and accurate mycobiome characterization [14]. Given these challenges, throughout this review we note the particular approach employed by each study to characterize mycobiota.

**Fig. 1:**
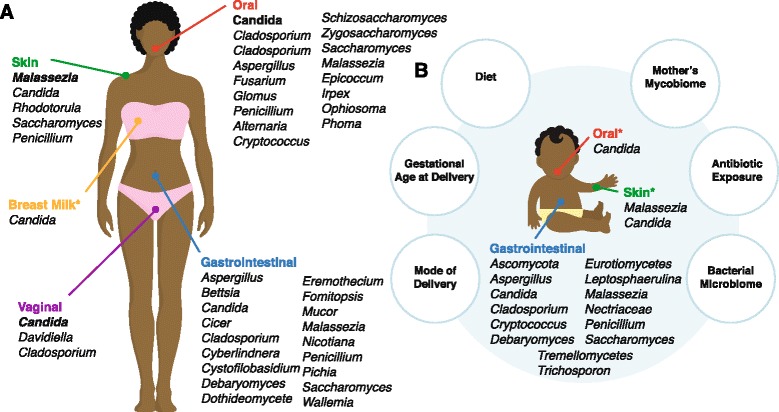
The mycobiome of infants is influenced by the mother’s mycobiome and other factors. Fungal genera associated with healthy adults (**a**) and infants (**b**). Body sites with an asterisk represent those that have only been characterized by culture or targeted PCR approaches; broad mycobiome surveys are lacking for these sites. Genera noted in bold type represent the dominant genus reported for that body site, as determined by broad mycobiome survey. Taxa listed are derived from targeted studies and broad survey approaches (≥1% of mycobiome) [2, 5, 14, 22–26, 28–39, 47, 51–54]. Also depicted are the major factors thought to influence the infant mycobiome

**Fig. 2:**
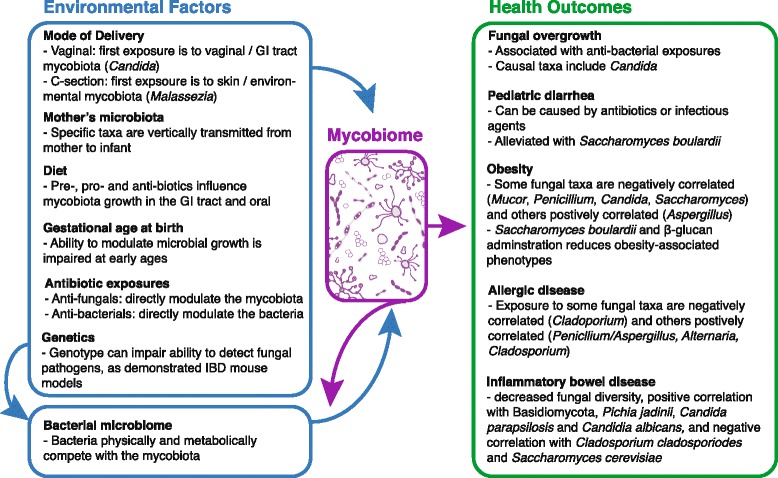
Factors influencing the infant mycobiome and mycobiome-associated health outcomes. Mode of delivery, mother’s microbiota, diet, gestational age at birth, and antibiotic exposure can influence the infant mycobiome. These factors can also impact the infant bacterial microbiome, which in turn shapes the mycobiome. The mycobiome has been noted as a source of fungal overgrowth [65–67], can modulate the bacterial microbiome [70], is implicated in inflammatory bowel disease [88–92], and has been associated with obesity [29, 76–80]. Exposure to fungi has been implicated in allergic disease development [82, 93] and beneficial fungi, such as *Saccharomyces boulardii*, can be used to alleviate pediatric diarrhea [84–86, 93]

Herein, we focus on the current state of mycobiota research in infants, including culture-based, targeted and broad survey-based genomic approaches, with a focus on two aspects of infant mycobiota research, namely factors influencing early-life mycobiota and potential links between early fungal exposures and health outcomes. Because research on the mycobiota of infants is limited, we consider the current state of fungal knowledge in the context of factors known to influence infant-associated bacterial microbiota, including mode of delivery, maternal mycobiota, feeding composition, environmental exposures (e.g., antibiotics), and gestational age at delivery [15–17]. Our findings are summarized in Figs. 1 and 2.

## Factors affecting infant mycobiota

### Birth mode

Whether an infant is born vaginally or through caesarean delivery (C-section) most drastically affects the composition of their associated bacterial communities over the first 6 months of life [18–21]. For example, in a study of 98 mother–infant pairs, vaginally-born infants had less fecal *Enterobacter*, *Haemophilus*, *Staphylococcus*, *Streptococcus*, and *Veillonella* species and had increased *Bacteroides*, *Bifidobacterium*, *Parabacteroides*, and *Escherichia* compared to infants born by C-section [20]. In this same study, vertical transmission of the mother’s fecal microbiota was likely the most significant contributor to the difference between vaginally and C-section-born infants, as 72% of bacteria colonizing feces of vaginally-born infants were present in their mother’s fecal microbiome, as compared to only 41% for C-section-born infants.

Vertical transmission of fungi from mother to infant has been most extensively studied with regard to the fungal species *Candida albicans*. In a study of very low birthweight infants, 24% of infants (*n* = 46) were colonized at a minimum of one site (oral cavity, rectum or groin) with *C. albicans* within 1 week of birth by the same *C. albicans* isolate present in either their mother’s vagina, rectum, skin or mouth, as determined by culturing and DNA fingerprinting (Fig. 1) [22]. Given this example of vertical transmission of one fungal taxon, it is likely that other members of the maternal vaginal mycobiota are also transferred to the infant. Therefore, by better understanding fungi that inhabit the birth canal of the mother, we may gain insight into which fungal taxa may be transferred to the infant.

Historically, studies have focused on the characterization of one fungal genus within the female genital tract, *Candida*, due to its importance as a cause of infection (vaginitis). In a study of *Candida* colonization of the vagina of asymptomatic women without a history of vaginal candidiasis, 28.8% of women were positive for *C. albicans* by targeted PCR analysis, demonstrating that *Candida* is a normal commensal of the female genital tract (Fig. 1). Of note, only 6.6% of these women were positive for *Candida* by culture, highlighting the limitations of fungal community analysis using culture-based approaches [5]. In another screen of asymptomatic women without a history of vaginal candidiasis, 40% (*n* = 52) of the sampled individuals carried at least one isolate of *Candida* (~90% of which were *C. albicans*) in their genital tracts (vulva or vagina), as determined by DNA fingerprinting of cultured *Candida* [23]. To our knowledge, there has only been one published broad survey of the vaginal mycobiome using the ITS region [24]; in this survey of healthy women (*n* = 251), *Candida* was the predominant genus and was present in 68% of samples, followed by *Davidiella*, *Cladosporium* and other less abundant fungi (Fig. 1). Of note, a large number of sequences in this study were not classified into a fungal taxonomic group, likely due to the under-representation of fungal sequences in currently available databases, as well as inconsistencies in the taxonomic classification of fungi. Nevertheless, the vaginal mycobiota likely plays an important role in the early colonization events of vaginally born infants.

Apart from *C. albicans*, mother–infant transmission has not been well studied for fungi. In a recent survey of four infants and their mothers using universal 18S rDNA primers [25], no amplicon was produced from DNA isolated from infant fecal samples at any time point, whereas the same samples were able to produce amplicons using universal 16S primers for bacteria. Fecal DNA from the mothers in this study produced 18S amplicons corresponding to *Blastocystis* (a parasite of the Stramenopile group), *Saccharomyces*, *Candida*, *Nicotiana*, and *Cicer*, among other fungal genera, leading the authors to conclude that the infant fecal samples contained no fungi. In contrast, in a study of very low birth weight infants, ITS2 amplicons were produced from the feces of the majority of infant samples (7 of 11) with the predominant genus being *Saccharomyces* (*S. cerevisiae*) followed by *Candida* (*C. albicans*, *C. glabrata*, *C. quercitrusa*, *C. diddensiae*, *C. parapsilosis*, and *C. tropicalis*), *Cladosporium* (*C. sphaerospermum* and *C. tenuissimum*), and *Cryptococcus* (*C. albidosimilas* and *C. podzolicus*) [26]. Similarly, in a study of 11 infant fecal samples using fungal-specific PCR along with ITS2-based sequence analysis, fungi were observed in all samples, with *C. albicans* being the predominant species followed by *C. parapsilosis*, *C. krusei*, and *Leptosphaerulina* [14]. *Penicillium*, *Aspergillus*, *Candida*, *Debaryomyces*, *Malassezia*, *Ascomycota*, *Eurotiomycetes*, *Tremellomycetes*, *Nectriaceae*, and *Trichosporon* were also observed in an ITS1 survey of infants under 2 years of age, and the presence of many specific taxa was confirmed by culture-based methods [27]. A longitudinal study of 14 infants also showed fungi to be present in the feces of all infants, at most time points, at a density of 10^4^–10^6^ rRNA genes/g of feces over the first 200 days of life [28]. This is in contrast to the 10^9^–10^10^ bacterial rRNA genes/g of feces reported for the same samples [28]. Despite one study reporting a lack of fungi in the infant GI tract, we conclude that most infants do harbor GI fungi (Fig. 1). Although fecal fungal RNA appears to be less abundant than bacterial RNA, fungal cells are considerably larger (100-fold) than bacterial cells. Thus, fungi contribute a substantial biomass to the fecal microbiota.

The extent of transfer of fungal communities from mother to infant remains unclear, but the overlap of the infant and the adult GI mycobiome does support the hypothesis of vertical mycobiota transmission. For example, in adults, fungi were detected in all fecal samples of 96 individuals by ITS sequencing, and the most abundant genera were *Saccharomyces*, *Candida*, and *Cladosporium* [2]. Similarly, in healthy controls (n = 12) from an ITS sequence-based survey of obese and lean individuals [29], *Mucor*, *Candida*, *Penicillium*, *Wallemia*, *Bettsia*, and *Cladoporium* were the predominant genera in feces, along with more minor members, and the healthy controls (*n* = 55) from a study of hepatitis-infected individuals were colonized with *Saccharomyces*, *Candida*, *Aspergillus*, *Malassezia*, *Penicillium*, and an uncharacterized fungus, as determined by 18S restriction fragment length polymorphism (RFLP) analysis [30]. The adult GI mycobiome, as characterized by ITS1 sequencing, has also been shown to contain *Candida*, *Penicillium*, *Aspergillus*, *Malassezia*, *Debaryomyces*, *Mucor*, *Eremothecium*, *Pichia*, and *Cyberlindnera* (healthy controls, *n* = 29) [31]. Further, *Saccharomyces* and *Penicillium* species were also detected by denaturing gradient gel electrophoresis and 18S rDNA sequence analysis of fecal samples from healthy adults, along with other fungal genera [32], and *Candida* species predominated in a study of 45 adult fecal samples using ITS sequencing [33]. In the latter study, only two fungal taxa were shared in a majority of samples, indicating an absence of core GI tract mycobiota [33]. Additionally, the observation of instability across longitudinal samples in the study further indicated the lack of a core mycobiota, although further testing with a larger sample size should be performed to confirm this hypothesis [33]. The fungal taxa shared across the infant and adult studies include *Candida*, *Saccharomyces*, and *Cladosporium*, but given the limited number of studies in infants and their small sample sizes, there are likely more fungal taxa that overlap between the two groups (Fig. 1). The observation of shared taxa between adults and infants provides support for the hypothesis of vertical transmission of GI mycobiota from mother to infant.

In C-section born infants, the bacterial microbiota of the skin and GI tract are more similar to those of the mother’s skin [21]. If this also holds true for the mycobiome, the skin and GI tract mycobiomes of C-section born infants would be expected to be dominated by *Malassezia* [34]. In a longitudinal study of infants that investigated skin colonization with *Malassezia* by *Malassezia*-specific PCR, two species (*M. restricta* and *M. globosa*, also observed on adult skin) were detected on the infant skin as early as the first day of life (89%), and abundance levels increased to that observed in adults by day 30 of life (Fig. 1) [35]. In addition, in the same study, vertical transmission of *Malassezia* from the skin of mothers to that of infants was confirmed by genotyping of the intergenic spacer region located downstream of the ITS1 and 2 regions. The targeted approach used in this study, however, prevents us from determining the proportion of *Malassezia* relative to other mycobiome members, warranting further exploration of the infant skin mycobiota with broad survey approaches.

To better understand the infant skin mycobiota as a community, we can consider adult studies that employ culture-dependent and independent methods. For example, in a study of ITS1 sequences from 14 skin sites of 10 healthy adults, the skin mycobiome, with the exception of the feet, was dominated by the genus *Malassezia* [34], consistent with the findings of other studies using culture and targeted PCR approaches [34–39]. Other members of the skin mycobiome, found either on a small number of people or in low abundance, included *Candida*, *Rhodotorula*, *Saccharomyces*, and *Penicillium*, along with several much less abundant fungi [34, 37]. For studies using ITS1 sequencing, however, it should be noted that such sequences are somewhat biased toward identification of basidiomycetous fungi, such as *Malassezia*, whereas ITS2 sequences favor identification of ascomycetous fungi such as most other human-associated fungi, including *Candida* [40]. Thus, it is possible that the ITS1-based approach used in the study by Findley et al. [34] was not sensitive enough to fully detect Ascomycetes present in the samples. Nevertheless, the vertical transmission of *Malassezia* from mother to infant further supports the hypothesis that birth mode impacts the mycobiota of the infant. Based on the studies mentioned above, we can hypothesize that infants born vaginally would have a higher proportion of *Candida* in their mycobiome given the *Candida*-predominated birth canal, and potentially have a more diverse mycobiome in comparison to those born by C-section given the exposure to the mother’s varied fecal mycobiota. Conversely, we can hypothesize that C-section infants, whose colonization source is often the mother’s skin, would be colonized by relatively higher amounts of *Malassezia* (Fig. 2).

### Diet

Diet composition, such as human milk or formula, strongly affects early infant bacterial microbiomes within the GI tract as well as in later infancy during the transition to solid foods [20, 41]. For example, breast-fed infants harbor more *Bifidobacteria* and *Labctobacilli* in their GI tracts in comparison to formula-fed infants [20, 28], likely due to the endogenous microbiome of human milk and human milk factors, such as oligosaccharides and immune proteins, that modulate the growth of certain bacteria. In several studies, many bacterial genera found in human milk (*Bifidobacterium*, *Bacteroides*, *Staphylococcus*, *Streptococcus*, *Pseudomonas*, *Lactobacillus*, and others) were also found in infant fecal samples [42–44]. Additionally, prebiotics in breast milk, such as human milk oligosaccharides, promote the preferential expansion of certain taxa such as *Bifidobacterium* species [45, 46]. Although the human milk mycobiome has yet to be characterized, we know that some fungi, such as *Candida* species, are found in milk from mothers with mammary candidiasis (67.4%, *n* = 46) as well as in asymptomatic controls (79.1%, *n* = 43) (Fig. 1) [47]. Human milk oligosaccharides have also been demonstrated to impact fungal virulence in vitro by decreasing the ability of *C. albicans* to invade intestinal epithelial cells [48]. Thus, it is rational to hypothesize that human milk also influences the infant GI mycobiota, although this remains to be tested.

Diet also affects the oral bacterial microbiota of infants [49], and this likely holds true for the mycobiota. In infants, oral colonization with fungi has only been studied using culture-dependent methods, with *Candida* species appearing to be the predominant fungi, although this finding may be biased because these species grow most easily on certain growth media. In one study of 100 healthy infants, 12% were orally colonized with *Candida* by 4 weeks of age and colonization prevalence rates did not change over the first 6 months of life [50]. Other studies also show oral *Candida* colonization rates to be low at birth, but to rise over time to adult carriage rates within the first year of life [51, 52]. The species of *Candida* currently reported as commensals of the oral cavity of infants include *C. albicans*, *C. parapsilosis*, *C. krusei*, *C. guillermondii*, *C. geocandidum*, and *C. tropicalis* [51, 52]. In adults, the oral mycobiota is reportedly diverse. For example, in one study using ITS2 sequencing to characterize oral mycobiomes, healthy individuals (*n* = 20) had a range of 9 to 23 fungal species within the oral cavity at a given time; the ‘core’ members of the oral community included *Candida*, *Cladosporium*, *Aspergillus*, *Fusarium*, *Glomus*, *Penicillium*, *Alternaria*, *Cryptococcus*, *Ophiosoma*, *Phoma*, *Schizosaccharomyces*, *Zygosaccharomyces*, and *Saccharomyces* [53]. Another study of adult saliva samples (*n* = 3) reported *Malassezia* as the dominant taxon, in addition to more minor mycobiome members such as *Epicoccum* or *Irpex* and others previously reported [54]. Therefore, given the diversity of the adult oral mycobiome, we can hypothesize that the oral mycobiome of infants likely contains other taxa in addition to *Candida*, and that the oral mycobiome of infants is likely dynamic, with changes especially noticeable as the infant transitions to a more adult-like diet (Fig. 2).

### Gestational age at delivery

Gestational age at delivery has been shown to impact the bacterial microbiota, including initial community differences and the pace of bacterial community maturation, but differences are often resolved by 2 years of age, when the bacterial community has reached an adult-like state [55–57]. For infants born at an early gestational age, the health impact of intestinal fungi is particularly significant, as the incidence of invasive, systemic candidiasis in these infants is approximately 10%, with an associated mortality rate of approximately 20% [58]. Susceptibility to invasive infection has been correlated with relative overgrowth of fungi, especially within the GI tract [8–10], as well as the presence of several predisposing clinical factors including a naïve immune system, bacterial dysbiosis due to antibiotic exposure, and use of parenteral nutrition, among other factors (Fig. 2) [59]. In an attempt to reduce rates of invasive candidiasis in this vulnerable population, prophylactic antifungals, such as nystatin and fluconazole, are often used in neonatal intensive care units. In particular, fluconazole has been shown to reduce *Candida* overgrowth at several body sites, including skin, the respiratory tract, and the GI tract, and its use has been associated with a decreased rate of invasive candidiasis in extremely low birth weight infants [60, 61]. Recently, the enteral administration of bacterial and fungal probiotics, such as *Lactobacillus reuteri*, *L. casei*, *L. rhamnosus*, *L. acidophilus*, *Streptococcus thermophilus*, *Bidiobacterium longum*, *B. bifidum*, *B. lactis*, and *S. boulardii*, have also been used to reduce invasive candidiasis [59, 60]; however, their efficacy remains unclear and their primary site of action is limited to the GI tract. Despite the administration of antifungals, fungal colonization still occurs in some infants, as demonstrated in a study of 11 extremely low birth weight infants, all of whom received an enteral anti-fungal treatment, nystatin, as well as antibacterial antibiotics [26]. Of these 11 infants, 7 produced ITS amplicons from their stool, with *Saccharomycetales* being the most abundant order, as well as *Candida* and *Cryptococcus* species. Thus, despite efforts to prevent fungal colonization, fungi maintain the ability to persist in the infant GI tract. In infants born at early gestational ages, beneficial fungi, such as *S. boulardii*, may help to regulate the growth of opportunistic fungal colonizers such as *Candida*. Additionally, given the association of bacterial dysbiosis with systemic candidiasis in infants born at an early gestational age, a robust bacterial community may play an important role in mycobiota regulation, as discussed below.

## Infant mycobiota health associations

### Antibiotic exposure and fungal overgrowth

The bacterial community of infants is significantly altered by exposure to antibiotics in both term and preterm infants [16, 62, 63]. For example, in a longitudinal study over the first 3 years of life, infants receiving multiple courses of antibiotics had immediate bacterial community changes following antibiotics and an overall less diverse fecal bacterial microbiome than those not given antibiotics [64]. Although most commonly used antibiotics do not directly act on fungi, anti-bacterial antibiotic exposure is associated with alterations to the mycobiota, such as increased rates of fungal colonization, fungal overgrowth, and changes in fungal community structure, as tested in mice [65]. In premature infants, exposure to third generation (broad-spectrum) cephalosporins is highly associated with an increased risk for both mucosal and invasive candidiasis [66], and in women, antibiotic exposure is associated with an increased incidence of fungal infections of the genitorurinary tract (Fig. 2) [67]. Recent studies have also reported that susceptibility to fungal overgrowth is primarily due to changes in the bacterial community following antibiotic exposure rather than immune status of the GI tract or the presence of specific fungal taxa [68, 69]. The proposed mechanisms of fungal colonization regulation by bacteria include physical restriction, resource competition and the production of anti-fungal molecules [68, 69]. Without these mechanisms of regulation, opportunistic fungi, such as *Candida*, can outgrow their occupied niche and initiate immune responses or cause acute infections, especially in immunocompromised individuals including premature infants.

The converse relationship that fungi affect bacterial colonization is also true, as recent animal experiments showed *C. albicans* colonization of the GI tract to be associated with altered bacterial repopulation following cefoperazone treatment, including increased *Enterococcus faecalis* levels and a lack of *Lactobacillus* recovery [70]. Specific bacterial and fungal taxa have also been shown to co-occur outside the scope of antimicrobial exposure. For example, in a study of stools from healthy adults, *Candida* was positively correlated with the presence of *Prevotella*, *Ruminococcus*, and *Methanobrevibacter* [2]*.* On adult skin, *Malassezia* was positively correlated with *Corynebacterium*, *Prevotella*, and *Propionibacterium* and negatively correlated with *Pseudomonas*, *Acinetobacter*, *Streptococcus*, and *Enhydrobacter* [71]. Additionally, certain fungal taxa are reported to regulate the growth of other fungi through the production of anti-fungal molecules. For example, the adult oral taxon, *Pichia*, is negatively correlated with the predominant oral taxon *Candida* and *Pichia*-produced factors isolated from growth media were able to prevent growth of *Candida*, *Fusarium*, and *Aspergillus* both in vitro and in mice [72]. Therefore, the fungal community is dynamic, self-regulating, and interacts with the bacterial community. Thus, future studies should include the analysis of both the bacterial and fungal components of the community in observational studies, as well as during experimental interventions (Fig. 2).

### Obesity

In adults and children, obesity is associated with altered bacterial microbiota and, in infants, administration of antibiotics (known modulators of the bacterial community) is also associated with obesity later in life [73–75]. Similarly, fecal mycobiomes of obese and lean adults are reported to differ, with the genera *Mucor* and *Penicillium* negatively correlating with increased measures of adiposity and weight gain, and the genus *Aspergillus* positively correlating with increased measures of adiposity and weight gain [29]. In children, obesity has been associated with lower abundances of *Candida* and *Saccharomyces* species, as analyzed by DNA fingerprinting and quantitative PCR [76]. However, the role of early fungal colonization of infants and obesity outcomes has not yet been studied. We hypothesize that, similar to bacteria, some early-life fungi will be beneficial and some detrimental to short- and long-term metabolic health. For example, administration of *S. boulardii* and fungal-specific molecules (β-glucan) to humans and animals have been shown to reduce obesity-related phenotypes [77–80]. Therefore, direct manipulation of obesity-related fungal taxa in early life should be explored as a potential preventative strategy against obesity.

### Allergic disease

Based on animal models and associations in humans, there are bacterial taxa that are associated with protection against allergic diseases later in life. For example, in a study of 319 infants, GI colonization with *Faecalibacterium*, *Lachnospira*, *Veillonella*, and *Rothia* was associated with a decreased risk of asthma and these same taxa were able to prevent airway inflammation in mice when used to colonize their GI tracts [81]. Whether there are key early-life fungal taxa that prevent allergic disease development remains unknown. However, a role for fungi in both allergy development and prevention was suggested by a study of 144 infants in whom exposure to airborne fungal spores from *Penicillium/Aspergillus* and *Alternaria* were positively associated with positive skin-prick tests for at least one allergen within a general allergy panel, whereas *Cladosporium* exposure was negatively associated with allergy development (Fig. 2). In addition, exposure to increased levels of environmental (bedroom) fungi during infancy were found to be negatively associated with later-childhood development of wheeze and asthma, whereas increased indoor *Aspergillus* and outdoor *Cladosporium* levels were positively associated with a later increased incidence of rhinitis [82]. Thus, although the specific taxa and clinical outcomes involved need further exploration, there is strong evidence that early life exposure to certain fungal taxa contribute to allergic disease (Fig. 2).

### Pediatric diarrhea

Diarrheal diseases are a significant cause of morbidity and mortality, accounting for approximately 760,000 deaths per year in children under the age of 5 [83]. The causes of diarrhea are varied, including antibiotic-associated and infectious diarrhea, amongst others. *S. boulardii* has been evaluated as a treatment and preventative strategy for many types of diarrhea, with mostly positive outcomes [84, 85]. For example, a meta-analysis of five clinical trials reported that *S. boulardii* is effective in preventing antibiotic-associated diarrhea in adults and children [86], and a separate meta-analysis also reported *S. boulardii* to be effective at reducing the duration of diarrhea in children with acute infectious gastroenteritis [87]. A third meta-analysis including 22 studies further concluded that *S. boulardii* can significantly reduce the duration of childhood diarrhea, whether caused by bacteria, viruses, or protozoa [85]. The mechanism whereby *S. boulardii* alleviates diarrhea is not yet clear, but animal models and in vitro work have shown *S. boulardii* to possess antimicrobial activities, inhibit bacterial toxins, promote barrier function of the intestinal epithelium, and elicit specific immune responses by the host [84]. Whether early exposure to *S. boulardii* is beneficial for the long-term health outcomes of infants remains to be tested, but the beneficial effect of *S. boulardii* on pediatric diarrhea is clear.

### Inflammatory bowel disease (IBD)

The dysbiotic microbial communities associated with IBD are becoming more defined, including fungi associated with disease development. In pediatric patients (aged 3–21 years), there is a clear IBD-associated fungal signature as analyzed by ITS1 sequencing, including increased relative abundances of *Pichia jadinii* and *Candida parapsilosis* and a decreased relative abundance of *Cladosporium cladosporiodes* (*n* = 32), as well as an overall decrease in fungal diversity within the stool of IBD patients as compared to controls (*n* = 90) [88]. An additional study also found pediatric IBD patients to have a fungal community different than healthy controls, including a predominance of Basidiomycota, although only a small portion of colonic biopsies yielded fungal amplicons (8 of 37 samples) [89]. These results are supported by ITS2 sequence analysis in a survey of adults with IBD that demonstrated an increased Basidiomycobiota/Ascomycota ratio, as well as an increased relative abundance of *Candida albicans* and decreased relative abundance of *Saccharomyces cerevisiae* in IBD patients (*n* = 235) as compared to controls (*n* = 38) [90]. While distinct gut fungal community structures are associated with IBD, it remains unclear as to whether microbial exposure or early colonization contributes to disease development. In a mouse model of chemically-induced colitis, fungi associated with IBD were capable of intestinal invasion only in animals deficient for the fungal pattern recognition receptor dectin-1 and not in wild-type animals. This suggests a causative role of host genetics, rather than a specific microbial community signature, in IBD [91]. Nevertheless, the fungal community appeared to contribute to disease severity, and future work regarding host-fungi interactions in early-life are needed to better understand IBD development and progression.

## Conclusion and future directions

Although there is a relatively small number of broad mycobiome surveys for infants, by considering them in aggregate we can begin to answer certain important biological questions regarding early-life mycobiota development. For example, the use of targeted studies has shown the vertical transmission of specific fungal taxa, such and *Candida* and *Malassezia*, from mother to infant [22, 35], and this type of mycobiota inheritance is worth exploring for all mycobiome members. Additionally, broad surveys of infant feces have captured snapshots of the dynamic fungal community in early life, and have begun to shed light on other fungal taxa inhabiting infants [14, 25, 26, 28]. The diversity and disease associations of adult mycobiota also provide further motivation to characterize infant mycobiota throughout development.

Future studies should focus on longitudinal tracking of the early-life mycobiota using mother–infant pairs while monitoring health outcomes, such as obesity, allergic disease, and acute fungal infections, well past the first year of life. The data generated from such studies would elucidate the impact of the mother’s mycobiota, and other environmental factors, on the infant mycobiota, and would document temporal changes of the mycobiome throughout development. Additionally, given the known interactions between the bacteria and fungi, future studies should include the analysis of both bacteria and fungi to better characterize their structural and functional relationships, as well as their combined effects on health outcomes. Furthermore, more research is needed to address the methodological challenges in generating fungal microbiome data, including sampling of low fungal biomass, generation of comprehensive and reliable reference databases, and improvement of informatics methods tailored to classifying fungal taxonomy and mycobiota function.
